# Effects of multiwalled carbon nanotube and *Bacillus atrophaeus* application on crop root zone thermal characteristics of saline farmland

**DOI:** 10.1016/j.heliyon.2023.e13510

**Published:** 2023-02-08

**Authors:** Yutian Zuo, Wenzhi Zeng, Chang Ao, Haorui Chen, Jiesheng Huang

**Affiliations:** a State Key Laboratory of Water Resources and Hydropower Engineering Science, Wuhan University, Wuhan, 430072, China; b Nanjing Hydraulic Research Institute, Nanjing, 210029, China; c State Key Laboratory of Simulation and Regulation of Water Cycle in River Basin, China Institute of Water Resources and Hydropower Research, Beijing, 100038, China

**Keywords:** Saline farmland, Crop root zone thermal characteristics, Multiwalled carbon nanotubes, *Bacillus atrophaeus*

## Abstract

Presently, the effects of crop roots on crop root zone thermal characteristics are poorly understood, and new fertilizers are rarely considered from the perspective of changing crop root zone thermal characteristics. This study explored the effect of applying two new fertilizers, multiwalled carbon nanotubes (MWCNTs) and *Bacillus atrophaeus* (B. atrophaeus), on the crop root zone thermal characteristics of saline farmland soils through in situ measurements. The results showed that MWCNTs and B. atrophaeus could indirectly affect crop root zone thermal characteristics by changing the crop root growth. Combined application of MWCNTs and B. atrophaeus could promote both to induce positive effects, promote crop root growth, and significantly alleviate the adverse effects of soil salinization. The thermal conductivity and heat capacity of the shallow root zone were reduced due to the presence of crop roots, while the opposite was true in the deep root zone. For example, the thermal conductivity of the 0–5 cm rich root zone in the MWCNT treatment was 0.8174 W m^−1^ ·K^−1^, and the thermal conductivity of the poor root zone was 13.42% higher than that of the rich root zone. MWCNTs and B. atrophaeus can also change the spatial distribution of soil moisture, soil salt, and soil particle size characteristics by influencing the root-soil interactions and indirectly affecting crop root zone thermal characteristics. In addition, MWCNTs and B. atrophaeus could directly affect the root zone thermal characteristics by changing the soil properties. The higher the soil salt content was, the more obvious the effect of the MWCNTs and B. atrophaeus on the crop root zone thermal characteristics. The thermal conductivity and heat capacity of the crop root zone were positively correlated with the soil moisture content, soil salt content and soil particle specific surface area and negatively correlated with the soil particle size and the fresh and dry root weights. In summary, MWCNTs and B. atrophaeus significantly affected crop root zone thermal characteristics directly and indirectly and could adjust the temperature of the crop root zone.

## 1 Introduction

At present, approximately 831 million hectares of land worldwide are affected by salinization. It is predicted that by 2050, more than 50% of the farmland on Earth will be affected by salinization [1]. Soil salinization will not only reduce crop yield and inhibit crop root growth but also lead to deterioration of crop metabolic function, reduction in crop energy demand, inhibition of crop cell division and other harmful effects [2]. In addition, soil salinization will also increase soil osmotic pressure and reduce soil water and fertilizer conservation capacity [3], reducing the activity of soil microorganisms and soil enzymes [4]. Therefore, alleviating the adverse effects of soil salinization has become an urgent technical problem. Researchers are continuously exploring how to make good use of existing saline farmland to alleviate the adverse effects of soil salinization on crops. Existing measures, such as fertilizer application and spring and winter irrigation, can improve the productivity of saline farmland and alleviate the adverse effects caused by soil salinization [5,6]. Although these measures can effectively increase crop yield and reduce the soil salt content in the crop root zone, they will inevitably damage the local ecological environment and easily cause adverse effects such as secondary salinization, soil compaction, water loss and soil erosion [7]. Therefore, it is essential to fundamentally improve the soil quality and reduce the fertilizer input so that the agricultural production system can sustainably develop in the fragile ecosystems. With the continuous change in global climate and increasingly frequent extreme weather, these adverse effects will become increasingly intense. By the end of this century, the global surface temperature will increase by 0.3–4.8 °C, which will lead to a rise in sea level, coastal land erosion and an increasing area of saline land (IPCC 2014). The global annual crop yield is predicted to decline by 2% every decade. With the continuous growth of the human population, food and land resources will become scarce, and the loss of land will pose a threat to human survival (IPCC 2014). Considering the impact of climate change on the availability of water resources in saline farmland and the fact that the change in root zone thermal characteristics will greatly affect the absorption and utilization of water resources by crops, the need for adjusting root zone thermal characteristics to improve the availability of water resources on saline farmland is more challenging.

Any biological and chemical processes in the soil, as well as the growth and development of crops and crop roots, must be carried out within a certain temperature range [8]. As early as the 19th century, scientists indicated the importance of soil thermal characteristics, which affect the allocation and distribution of energy on the soil surface and in the soil profile and are of great significance in various studies and applications [9]. Many studies have shown that soil thermal properties can be used to predict soil temperature [10], soil moisture characteristic curves [11], soil physical characteristics [12], soil organic carbon content [13], soil texture [14], and greenhouse gas emissions [15] and improve soil microbial activity [16]. Thermal characteristics are also of great significance in the engineering design and evaluation of soil thermal states and degrees of frost heave [17]. Soil thermal characteristics are strongly affected by soil moisture content, soil texture, soil structure, soil organic carbon content, soil bulk density, air-filled porosity and soil particle size characteristics [18,19]. In addition, crop roots also play an important role in the construction of the soil structure through a series of mechanical, biochemical and biological processes. Changes in the soil structure and the differences between the characteristics of crop roots and soil particles must lead to changes in the crop root zone thermal characteristics [20]. The volume of soil in the root zone accounts for more than 50% of the total volume of farmland soil, and it is necessary to study the crop root zone thermal characteristics to regulate the temperature of the crop root zone [21]. The existing relevant studies are mostly based on soil thermal characteristics, and few researchers consider the influence of crop roots on the root zone thermal characteristics, as well as the response mechanisms among the crop root zone thermal characteristics, soil properties and crop root characteristics. Therefore, finding a new type of fertilizer that can adjust the root zone thermal characteristics of saline farmland, alleviate the adverse effects of soil salinization on crops, reduce fertilizer input, and promote sustainable agricultural development is a hot topic in current agricultural production research.

According to recent domestic and international research, nanotechnology and microbial technology have promoted sustainable agricultural development in terms of fertilizer delivery, genetic modification and pest control [22,23]. However, according to previous studies, nanomaterials or strains were often applied separately to improve soil, and few studies combined the two and used to improve the crop root zone thermal characteristics. Based on the above background, this study selected two new fertilizers that are suitable for saline farmland soils, namely, multiwalled carbon nanotubes [24] and *Bacillus atrophaeus* [25], referred to as MWCNT and B. atrophaeus, respectively. These fertilizers have been shown to reduce fertilizer input, improve soil physical and chemical properties, promote seed germination, and increase crop yield and fruit quality [26,27]. This study used the saline soil in the autonomous county of Yanqi Hui, Bayingol Mongol Autonomous Prefecture, Xinjiang, China, as the research object. A pot simulation experiment was carried out to explore (1) the effects of applying MWCNTs and B. atrophaeus on the crop root zone thermal characteristics of saline farmland soils, (2) the effects of applying MWCNTs and B. atrophaeus on the soil properties and crop root properties of saline farmland soils, and (3) an analysis of the response mechanisms among crop root zone thermal characteristics, soil properties and crop root characteristics. This study has important scientific significance for regulating the crop root zone thermal characteristics and improving the crop root zone soil quality, crop productivity and sustainable agricultural development of saline farmland and lays a solid theoretical basis for the large-scale application of MWCNTs and B. atrophaeus on saline farmland in the future.

## 2 Materials and methods

### 2.1 Test materials

**Fig. 1:**
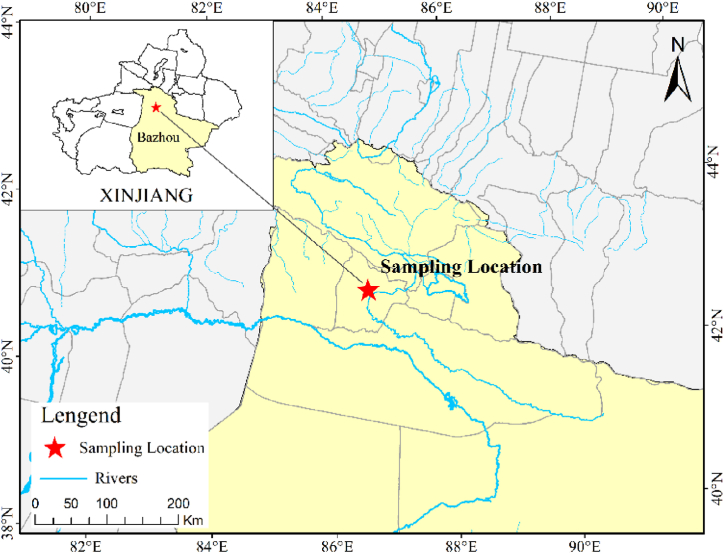
Map of the study region.

The soil samples were collected from the autonomous county of Yanqi Hui, Bayingol Mongol Autonomous Prefecture, Xinjiang, China, at a geographical location of approximately 41°45′N, 85°44′E. A map of the study region is shown in Fig. 1. The annual sunshine duration in this area is approximately 2500–3000 h, the annual average temperature is approximately 5–10 °C, precipitation is scarce (approximately 50–250 mm per year), and the potential evaporation demand is large (approximately >1000 mm per year), resulting in serious soil salinization [28]. The soil is a sandy loam and belongs to the Terric Anthrosols (Loamic, Salic) according to the IUSS working group WRB classification [29]. In the experimental area, the stratified random multipoint mixing method was used to collect soil samples, and undisturbed soil obtained from each stratified layer was put into a bag and sealed until use. The selected MWCNTs were purchased from the Chengdu Organic Chemical Co., Ltd., Chinese Academy of Sciences (https://www.cocc.cn/). The purity was >98%, the outer diameter was 5–15 nm, the inner diameter was 2–5 nm, the length was 0.5–2 μm, and the surface area was >350 m^2^/g. The MWCNT solutions were dispersed and suspended in ultrapure water with a 44 kHz ultrasound for 12 h in an ultrasonic cleaner until the final concentration was 75 mg/L for seed soaking and root irrigation. Seed soaking refers to the soaking of sterile seeds in a 75 mg/L MWCNT suspension for 6 h before sowing, and root irrigation refers to a single irrigation with 10 ml of suspension of the above concentration per pot to crop roots during the first irrigation. The selected B. atrophaeus was purchased from the China Center for Type Culture Collection (https://cctcc.whu.edu.cn/), with short stems, multi-cluster arrangement, aerobic conditions and no need for light. The B. atrophaeus was inoculated with 10 ml of an obacterial solution with an OD600 of 0.8 per pot, which was irrigated to the crop roots once during the first irrigation.

### 2.2 Experimental scheme and research methods

The experiment was carried out in the greenhouse of the Irrigation and Drainage Comprehensive Test Field of the State Key Laboratory of Water Resources and Hydropower Engineering Science of Wuhan University; the experiment included 6 treatments: the control group (CK treatment); seeds soaked with MWCNT suspension (CM treatment); seeds soaked with MWCNT suspension and using MWCNT suspension to irrigate crop roots (CR treatment); inoculation with B. atrophaeus (BK treatment); inoculation with B. atrophaeus and seeds soaked with MWCNT suspension (BM treatment); inoculation with B. atrophaeus, seeds soaked with MWCNT suspension and the use of MWCNT suspension to irrigate crop roots (BR treatment). Each of the above treatments was established for two soil salt content conditions, namely, a high salt condition (denoted by an H prefix, the conductivity of the 1:5 soil‒water extract was 1200 μs/cm) and a low salt condition (denoted by an L prefix, the conductivity of the 1:5 soil‒water extract was 420 μs/cm); in total, there were 12 test treatments with five replicates for each treatment. The planting container was a cylindrical container with a diameter of 20 cm and a height of 20 cm. To imitate the state of the tilth soil layer, relevant stratified filling of soil columns with different soil layers in the field was conducted by the plate compaction method; the initial soil bulk density of each treatment was 1.35 g/cm^3^, and the initial water content of each treatment was 0.25 cm^3^/cm^3^ [23]. Tests were performed under aseptic conditions to cultivate maize seeds (*Zea mays* L.) after 7 days, seedlings that had grown consistent were selected to be transplanted, and tests were conducted in the 30-day growth period after transplanting. During the experiment, natural light irradiation was used to simulate the actual growth of the maize, and purified water was used for irrigation to maintain the normal growth of the crops and ensure the same total water input for all treatments, i.e., the same total moisture of all treatments. After the experiment, the SH-3 probe of a thermal characteristic analyzer (TEMPOS, METER Company, USA) was used to measure the crop root zone thermal characteristics (thermal conductivity, thermal capacity, and thermal diffusivity) in different layers (0–5 cm, 5–10 cm, 10–15 cm) at 2 cm from the edge of the planting container (poor root zone) and 2 cm from the crop (rich root zone) in situ. The probe was calibrated before the measurement and vertically inserted. At the same time, the soil moisture content was measured by the drying method. The soil organic carbon content was measured by an organic carbon analyzer (TOC-5500, Shanghai Metash Instrument, China). A conductivity meter (HQ40d, HACH Company, USA) was used to measure the electrical conductivity of the 1:5 soil‒water extract, and the soil salt content was reflected by the soil electrical conductivity. Soil particle size characteristics were measured by a particle size analyzer (S3500, Microtrac Inc., USA). After the above steps, the crop roots were separated from the soil, and the fresh and dry weights of the crop roots were measured. The detailed measurement methods are described in Ref. [30].

### 2.3 Statistical analysis

SPSS 22.0 and Origin 2019 were used for analyzing data and plotting Figures and tables. The means and standard deviations for all the parameters were calculated from at least three replicates. To compare differences among the treatments, one-way analysis of variance (one-way ANOVA) and the least significant difference (LSD) test were used at p < 0.05. Spearman's correlation coefficient was calculated to estimate the correlation relationships among crop root zone thermal characteristics, soil properties and crop root characteristics.

## 3 Results and analysis

### 3.1 Crop root zone thermal characteristics

#### 3.1.1 Thermal conductivity

**Fig. 2:**
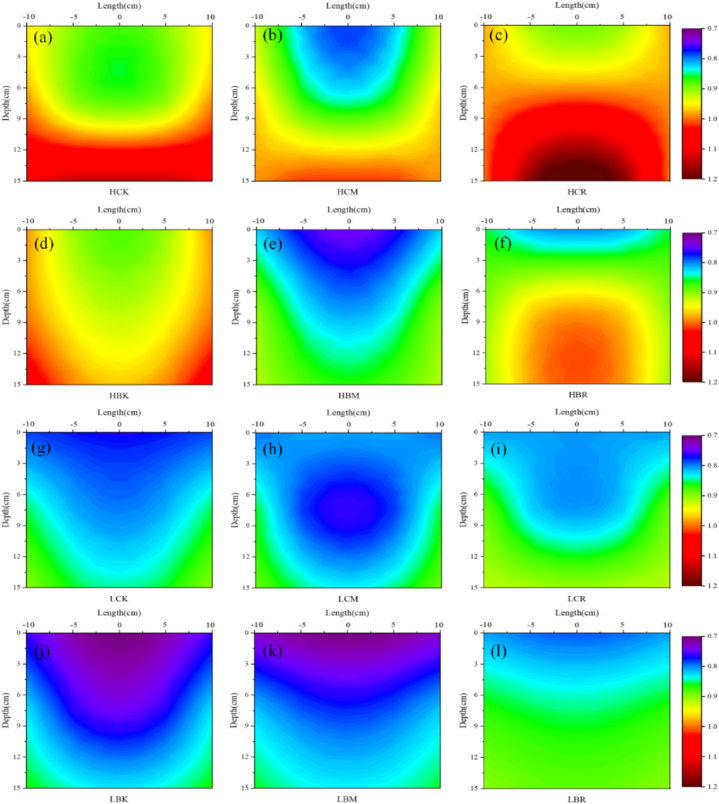
Spatial distribution of the thermal conductivity (W·m^−1^ ·K^−1^) of the root zone during sampling (a: HCK; b: HCM; c: HCR; d: HBK; e: HBM; f: HBR; g: LCK; h: LCM; i: LCR; j: LBK; k: LBM; l: LBR; In each Figure, the right half is the interpolated result of the measured data and the left half is the mirror image of the right half, showing the assumed symmetrical root distribution).

The spatial distribution of the thermal conductivity of the root zone during sampling is shown in Fig. 2(a - l). Each treatment had a significant impact on the thermal conductivity of the crop root zone, and there was a significant difference between the rich root zone and the poor root zone.

Fig. 2(a - l) show that the root zone thermal conductivity gradually increases with increasing soil depth. The thermal conductivity of the root zone under high salt conditions was higher than that under low salt conditions. For example, the average thermal conductivity of the LCK treatment was 0.8315 W m^−1^ ·K^−1^, and that of the HCK treatment increased by 20.10% in comparison. The thermal conductivity of the shallow rich root zone was lower than that of the shallow poor root zone. For example, the thermal conductivity of the 0–5 cm rich root zone in the HCM treatment was 0.8174 W m^−1^ ·K^−1^, and the thermal conductivity of the poor root zone was 13.42% higher than that of the rich root zone. The thermal conductivity of the deep rich root zone is generally lower than that of the deep poor root zone; however, the opposite is true in the CR and BR treatments, which may be related to the crop root characteristics and the MWCNT application through root irrigation. In addition, regardless of the high or low salt conditions, the CR treatment significantly increased the average thermal conductivity of the root zone, and the CM treatment had no significant effect on the average thermal conductivity of the root zone. The application of B. atrophaeus significantly reduced the average thermal conductivity of the root zone, and the combined application of B. atrophaeus and MWCNTs (BR treatment) slowed the decreasing trend of thermal conductivity.

#### 3.1.2 Heat capacity

**Fig. 3:**
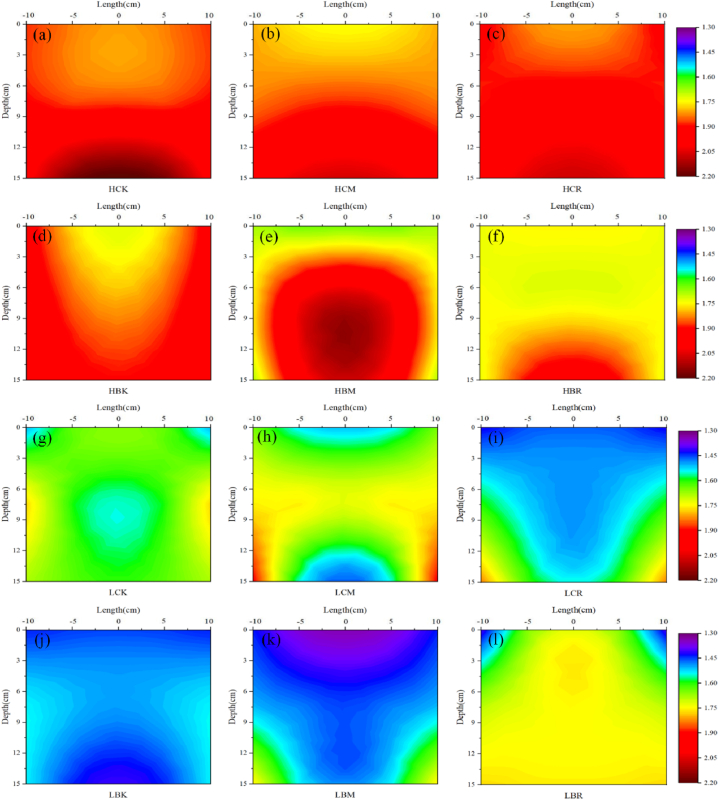
Spatial distribution of the heat capacity (J·cm^−3^·K^−1^) of the root zone during sampling (a: HCK; b: HCM; c: HCR; d: HBK; e: HBM; f: HBR; g: LCK; h: LCM; i: LCR; j: LBK; k: LBM; l: LBR; In each Figure, the right half is the interpolated result of the measured data, and the left half is the mirror image of the right half, showing the assumed symmetrical root distribution).

The spatial distribution of the root zone heat capacity during sampling is shown in Fig. 3(a - l). Each treatment had a significant impact on the heat capacity of the root zone, and there was a significant difference between the rich root zone and the poor root zone.

Fig. 3(a - l) show that generally, with increasing soil depth, the heat capacity of the root zone gradually increases (except for the LCM and LBR treatments). The average heat capacity of the root zone under high salt conditions was higher than that under low salt conditions. For example, the average heat capacity of the LCK treatment was 1.6386 J cm^−3^ K^−1^, and that in the HCK treatment increased by 18.47% in comparison. The heat capacity of the shallow root rich zone was less than that of the root rich zone. For example, the heat capacity of the 0–5 cm rich root zone in the HBK treatment was 1.771 J cm^−3^ K^−1^, and the heat capacity of the poor root zone was 9.43% higher than that of the rich root zone. The heat capacity of the deep rich root zone was greater than that of the poor root zone under high salt conditions, while the opposite was true under low salt conditions, which may be related to the spatial distribution of salt in the soil and the crop root characteristics. In addition, under high salt conditions, the average heat capacity of each treatment was lower than that of the HCK treatment. The HCM and HCR treatments had no significant effect on the average heat capacity of the root zone. The HBR treatment significantly reduced the heat capacity of the root zone. However, under low salt conditions, only the LBR treatment significantly increased the average heat capacity of the root zone, and only the LCM treatment had no significant effect on the average heat capacity of the root zone. The average heat capacity was significantly lower in the other treatments than in the LCK treatment.

#### 3.1.3 Thermal diffusivity

**Fig. 4:**
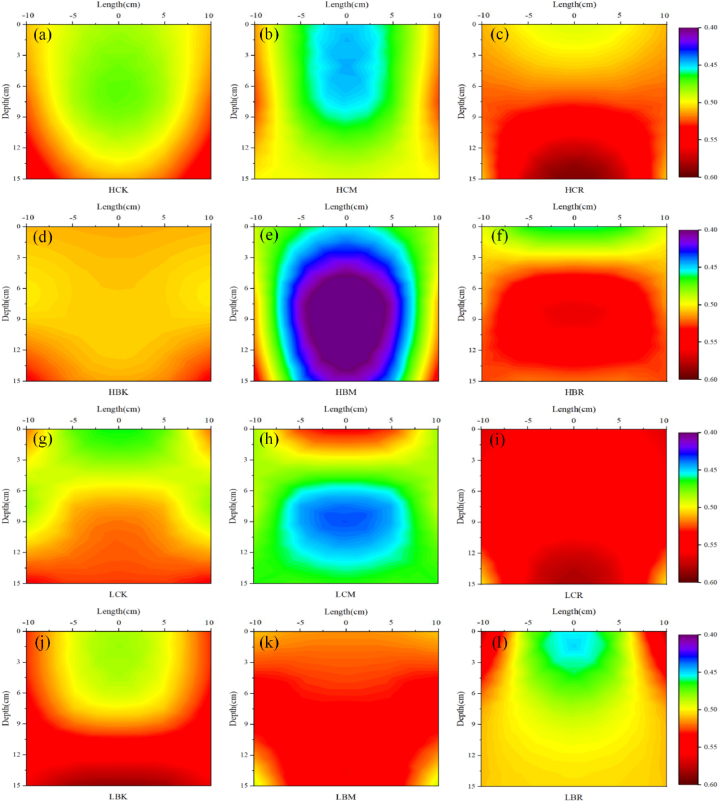
Spatial distribution of the thermal diffusivity (m^2^·s^−1^) of the root zone during sampling (a: HCK; b: HCM; c: HCR; d: HBK; e: HBM; f: HBR; g: LCK; h: LCM; i: LCR; j: LBK; k: LBM; l: LBR; In each Figure, the right half is the interpolated result of the measured data, and the left half is the mirror image of the right half, showing the assumed symmetrical root distribution).

The spatial distribution of the root zone thermal diffusivity during sampling is shown in Fig. 4(a - l). Each treatment had a significant impact on the root zone thermal diffusivity, and there was a significant difference between the rich root zone and the poor root zone.

Fig. 4(a - l) show that with increasing soil depth, the root zone thermal diffusivity of the CK, BK, CR, BR, LBM and HCM treatments increased. The thermal diffusivity of the other treatments (HBM and LCM treatments) decreased with increasing soil depth, especially in the rich root zone. The difference in root zone thermal diffusivity between high salt conditions and low salt conditions was inconsistent. The average root zone thermal diffusivity of the CR and BK treatments under high salt conditions was lower than that under low salt conditions. For example, the average thermal diffusivity of the LCR treatment was 0.5481 m^2^ s^−1^, which was 4.56% less than that of the LCR treatment. However, the average root zone thermal diffusivity of BM and BR treatments under high salt conditions was higher than that under low salt conditions. In addition, the difference in the average root zone thermal diffusivity between the high salt condition and the low salt condition in the CK treatment was not significant. Notably, the CR treatment significantly increased the average thermal diffusivity of the root zone under both high and low salt conditions.

### 3.2 Changes in soil properties

#### 3.2.1 Soil moisture content

**Fig. 5:**
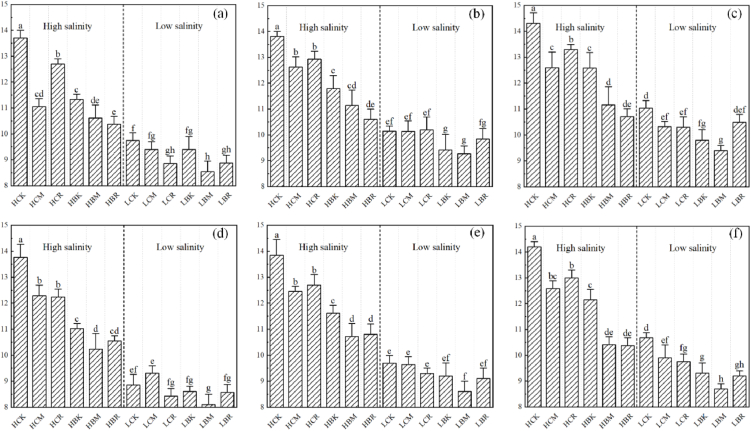
Spatial distribution of soil moisture (%) in the root zone (a: 0–5 cm layer of the poor root zone; b: 5–10 cm layer of the poor root zone; c: 10–15 cm layer of the poor root zone; d: 0–5 cm layer of the rich root zone; e: 5–10 cm layer of the rich root zone; f: 10–15 cm layer of the rich root zone; lowercase letters show significant differences among the treatments, p < 0.05).

Soil moisture content is an important characteristic that affects crop root zone thermal characteristics. The spatial distribution of the soil moisture content in the root zone is shown in Fig. 5(a–f). Each treatment significantly affected the spatial distribution of the soil moisture content in the root zone. In this study, the soil moisture evaporation was assumed to be the same in all treatments, so the soil moisture content could be used to represent the crop water consumption.

Fig. 5(a–f) show that the soil moisture content under high salt conditions is higher than that under low salt conditions, and the soil moisture content gradually increases with increasing soil depth. The soil moisture content in the rich root zone was less than that in the poor root zone. For example, the average moisture content in the rich root zone under the LCK treatment was 9.74%, which was 6.19% higher than that in the rich root zone. As the crop root absorbs moisture from the soil near the crop root, the soil moisture laterally transfers due to the potential energy difference, thus it also represents the root density of each treatment to a certain extent. For example, the difference between the average moisture content of the poor root zone and rich root zone in the HBM treatment increased by 67.15% compared with that in the HBK treatment, which indicated that the crop root density in the HBM treatment was greater than that in the HBK treatment. In addition, regardless of the high salt or low salt conditions, the soil moisture content of each modified treatment was lower than that of the CK treatment, which meant that each treatment had a role in promoting crop growth. The soil moisture content in all layers of the LBM treatment was the lowest of all treatments, of which the soil moisture content of the 0–5 cm rich root zone was the lowest, reaching 8.11%. Compared with the application of MWCNTs alone, the application of B. atrophaeus alone significantly reduced the soil moisture content, and the combination of both fertilizers (BM, BR treatment) promoted a reduction trend in soil moisture content. B. atrophaeus application promoted crop growth better than did the MWCNT application, and combined application had a positive effect. Notably, the HCR treatment significantly increased the soil moisture content compared with the other modified treatments.

#### 3.2.2 Soil salt content

**Fig. 6:**
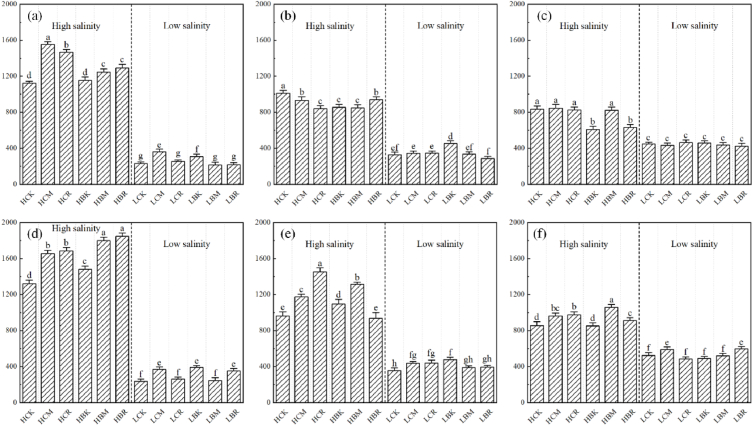
Changes in root zone soil conductivity (μS/cm) (a: 0–5 cm layer of the poor root zone; b: 5–10 cm layer of the poor root zone; c: 10–15 cm layer of the poor root zone; d: 0–5 cm layer of the rich root zone; e: 5–10 cm layer of the rich root zone; f: 10–15 cm layer of the rich root zone; lowercase letters show significant differences among the treatments, p < 0.05).

Soil salt content is an important characteristic that affects root zone thermal characteristics. In this study, soil conductivity was used to reflect the salt content of the root zone soil. The conductivity of the root zone soil is shown in Fig. 6(a–f). Each treatment significantly affected the conductivity of the root zone soil.

Fig. 6(a–f) show that with increasing soil depth, the soil salt content under high salt conditions decreases, while the soil salt content under low salt conditions increases, and the difference in soil salt content between treatments decreases. The reason for the difference in the vertical distribution of soil salt content under the two soil salt conditions is that the salt migrates upward with soil water and leaches downward with irrigation. Under high salt conditions, HBM treatment significantly increased the deep soil salt content compared with the other modified treatments. In addition, with the moisture absorption of the crop roots, the soil salt will migrate to the vicinity of the crop root with the moisture driven by the potential energy difference, resulting in a higher soil salt content in the rich root zone than in the poor root zone. The lateral migration rate of soil salt is related to the crop root characteristics. The more developed the crop root is, the greater the lateral migration of the soil salt. For example, the average soil conductivity of the HBM treatment in the rich root zone was 1390.50 μS/cm and was 30.09% lower in the poor root zone than in the rich root zone. The effect of applying MWCNTs on soil salt migration in the root zone was greater than that of applying B. atrophaeus, and the combined application of MWCNTs and B. atrophaeus (HBM and HBR treatments) significantly increased the lateral migration of soil salt.

#### 3.2.3 Soil particle size characteristics

Soil particle size characteristics are important characteristics that affect the crop root zone thermal characteristics. The soil particle size characteristics during sampling are shown in Table S1. Each treatment significantly affected the soil particle size characteristics of the root zone.

Table S1 shows that soil salt will reduce the soil particle size and soil particle size abundance and increase the specific surface area and soil clay content. The MV of the BM treatment was not significantly different between the high salt and low salt conditions, and the difference was only 3.61 μm. The HBR treatment significantly increased the specific surface area of soil particles under high salt conditions, while the LBR treatment had no significant effect on the specific surface area of soil particles under low salt conditions. The effect of each treatment on Dia was not significant, and the soil particle size richness could not be significantly increased. Under high salt conditions, the HBM and HBR treatments significantly reduced the soil clay content; for example, the clay content of the HBR treatment was 7.47%, which was 11.70% lower than that of the HCK treatment. In general, the HBR treatment significantly affected the soil particle size characteristics under high salt conditions, and the effect of each treatment on the soil particle size under high salt conditions was higher than that under low salt conditions. There was no significant difference in the soil particle size characteristics among the treatments under low salt conditions.

#### 3.2.4 Soil organic carbon content

**Fig. 7:**
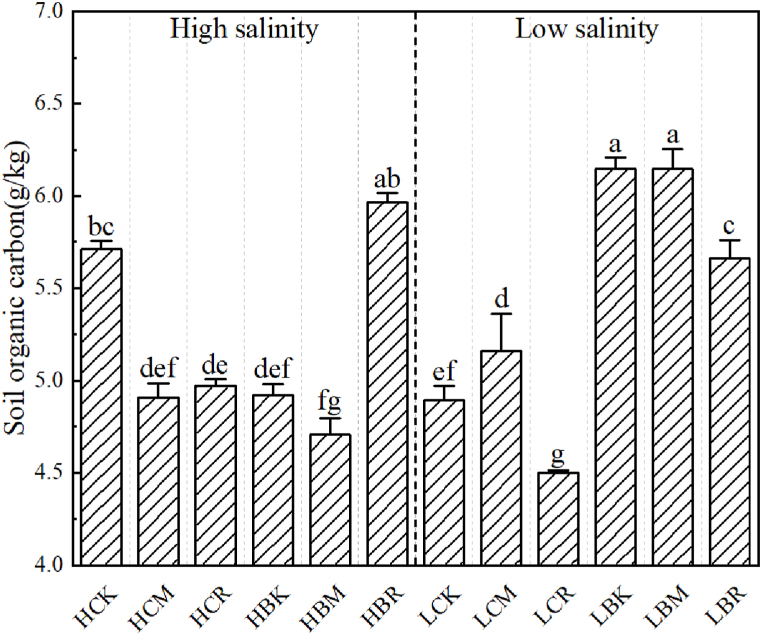
Changes in soil organic carbon content (lowercase letters show significant differences among the treatments p < 0.05).

Soil organic carbon content is an important factor that affects root zone thermal characteristics. The soil organic carbon content of each treatment during sampling is shown in Fig. 7. Each treatment significantly affected the soil organic carbon content.

Fig. 7 shows that under high salt conditions, the treatments significantly reduced the soil organic carbon content, except in the HBR treatment, in which the soil organic carbon content was not significantly different from that of the HCK treatment. For example, the organic carbon content of the HCK treatment was 5.71 g/kg, and that in the HCM treatment decreased by 14.06% in comparison. Under low salt conditions, all the treatments significantly increased the soil organic carbon content, except in the LCR treatment, in which the soil organic carbon content was significantly reduced compared to that in the LCK treatment. For example, the organic carbon content of the LCK treatment was 4.90 g/kg, and that in the LBM treatment increased by 25.49% in comparison. In addition, there was no notable pattern between the soil total salt content and soil organic carbon content.

### 3.3 Crop root characteristics

**Fig. 8:**
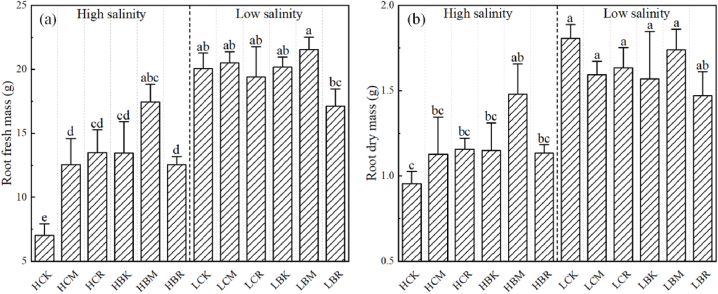
Changes in crop root characteristics (a: Crop root fresh weight; b: Crop root dry weight; lowercase letters show significant differences among the treatments, p < 0.05).

In this study, two crop root indicators were selected to represent the crop root characteristics. The fresh and dry weights of the crop roots are shown in Fig. 8(a and b). Each treatment significantly affected the crop root characteristics.

Fig. 8(a and b) show that soil salt significantly reduces the fresh and dry weights of the crop roots and inhibits their growth. For example, the fresh weight of the crop roots in the HCK treatment was 7.02 g, and that in the LCK treatment increased by 64.97% in comparison. Under high salt conditions, the crop root fresh weight of all the treatments increased compared with that of the HCK treatment, and the HBM treatment had the best improvement effect. Compared with the HCK treatment, the crop root fresh weight in the HBM treatment increased by 59.79%. Except for in the HBM treatment, which significantly increased the crop root dry weight, the effects of each treatment on the crop root dry weight were not significant. Under low salt conditions, all the treatments had no significant effect on the crop root characteristics. In addition, under both high and low salt conditions, each treatment significantly increased the moisture content of the crop roots compared with the CK treatment. Because the crop root was still in a growth period, the high moisture content of the crop root represented a high proportion of new roots of the crop root system, more frequent moisture exchange with the soil environment, and stronger functionality of the crop root system.

### 3.4 Correlation analysis of crop root zone thermal characteristics, soil properties and crop root characteristics

**Fig. 9:**
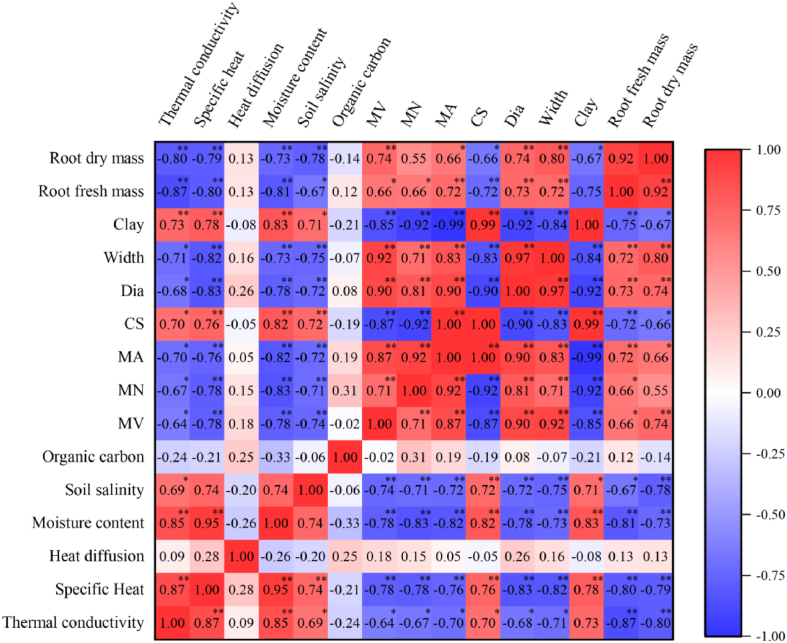
The correlation coefficient matrix of root zone thermal characteristics, soil properties and crop root characteristics (the asterisks indicate that there is a significant correlation among the root zone thermal characteristics, soil properties and crop root characteristics: p < 0.05*, p < 0.01**).

The correlation coefficient matrix of root zone thermal characteristics, soil properties and crop root characteristics are shown in Fig. 9.

Fig. 9 shows that the root zone thermal conductivity is significantly positively correlated with the soil moisture content, soil salt content, CS and soil clay content and negatively correlated with the MV, MN, MA, Dia, Width, and the fresh and dry weight of the crop roots but not significantly correlated with the soil organic carbon content. The correlations among the heat capacity of the root zone, the soil properties and the crop root characteristics were the same as those for the thermal conductivity of the root zone, while the thermal diffusivity of the root zone had no significant correlation with the soil properties and crop root properties. The highest correlation coefficient was 0.95 between the root zone heat capacity and soil moisture content. In addition, there was also a good correlation between the crop root characteristics and soil properties. The crop root fresh weight was significantly positively correlated with the soil moisture content, MV, MN, MA, Dia, and Width were negatively correlated with the soil salt content, CS, and soil clay content. The correlation between the crop root dry weight and the soil properties was consistent with that of the crop root fresh weight. There was a strong correlation among the root zone thermal characteristics, soil properties and crop root characteristics, which proves that crop roots cannot be ignored in the study of root zone thermal characteristics.

## 4 Discussion

The results above indicate that applying MWCNTs and B. atrophaeus not only directly affects the crop root zone thermal characteristics of saline farmland by changing their soil properties but also indirectly affects those characteristics by changing the crop root growth and root-soil interactions.

### 4.1 Indirect impacts

First, the application of MWCNTs and B. atrophaeus significantly affected crop root growth in saline farmland soils. Because crops usually need to be irrigated and irrigation is usually applied along with fertilizer, MWCNTs applied by root irrigation, the simplest and most attractive treatment method, was selected in this study. Zhai et al. found that most MWCNTs applied by root irrigation remained in the soil or attached to the root surface, which could not only improve the root zone soil properties but also promote the growth of crop roots, thereby significantly affecting the crop root zone thermal characteristics [31]. Khodakovskaya et al. also found that a small number of MWCNTs could enter crop cells to upregulate the genes involved in cell division, cell wall formation, and water transport and promote the growth of crops and crop roots [32]. At the same time, due to the special structure and inherent water transmission characteristics of MWCNTs, maize seeds soaked in MWCNT solutions can well regulate the moisture flow during seed soaking, achieve a balance between excessive blockage of the seed coat and complete free flow of water, and slow the rate of penetration and electrolyte leakage, thus significantly improving the seed germination rate and greatly improving the ability of the crop to resist adverse effects during the growth period [33]. Dasgupta-Schubert et al. also showed that the moisture content of seeds soaked with MWCNTs increased, and that they were more sensitive to changes in the MWCNTs themselves and the medium in which the MWCNTs were dispersed [34]. In addition, B. atrophaeus can improve crop fruit quality and crop antioxidant levels under salt stress, increase crop yield, and alleviate the adverse effects of salt stress on crop growth, thus promoting sustainable agricultural development [35]. Banerjee et al. also showed that the application of B. atrophaeus, which has adapted to salinization conditions, can increase crop survival, have a positive impact on the development of plants under stress, significantly improve the germination rate of seeds under saline conditions, and increase the root length of maize [22].

This shows that these two new fertilizers can effectively promote crop root growth under salt stress and indirectly affect the crop root zone thermal characteristics. There are great differences in the thermal characteristics, moisture content and hydraulic properties between the crop roots and the surrounding soil, so the presence of roots will certainly affect the crop root zone thermal characteristics. Haruna and Anderson found that when the crop root density was greater than a critical value, the soil became deformed, the crop root zone soil porosity increased, the soil bulk density decreased, and the proportion of the gas phase in the soil increased [36]. Whalley et al. also found the same results; compared with that of rootless soil, the soil total porosity increased in the maize root zone, with more macropores and fewer mesopores [37]. The soil deformation caused by crop roots mostly acts on shallow soil, and in general, the thermal conductivity and heat capacity of the air are lower than those of soil particles and moisture. MWCNTs and B. atrophaeus can reduce the thermal conductivity and heat capacity of the shallow crop root zone by promoting the growth of crop roots, increasing the deformation degree of the shallow soil porosity. This contrasts with the conclusions of Fu et al. who found that the presence of crop roots increased the thermal conductivity and heat capacity of the soil [38]. The same rule was observed in the deep soil in this study; for example, the heat capacity and thermal diffusivity of the deep rich root zone in the HBR treatment were greater than those of the poor root zone (Fig. 3, Fig. 4). Because the moisture content of crop roots is usually higher than that of surrounding soil particles, the heat capacity of water is much higher than that of dry roots and soil particles [39,40]. Moreover, the mucus secreted by maize roots can replace the air in the soil, reduce the soil surface tension, increase the soil matrix potential, improve the soil water retention in the crop root zone, stabilize the soil structure, increase the soil moisture content, and increase the thermal conductivity and heat capacity of the crop root zone [37,41]. MWCNT and B. atrophaeus addition can change the deep crop root zone soil proportion by promoting crop root growth, thereby increasing the thermal conductivity and heat capacity of the deep crop root zone. Therefore, the application of MWCNTs and B. atrophaeus will significantly promote the growth of crop roots, and their combined application in this study also achieved good results, promoting each other and playing a positive role, thereby reducing the thermal conductivity and heat capacity of the shallow crop root zone and increasing the thermal conductivity and heat capacity of the deep crop root zone.

Moreover, MWCNT and B. atrophaeus application also affects the spatial distribution of the soil moisture by changing the root-soil interactions, thereby indirectly affecting crop root zone thermal characteristics [42]. Soil moisture content is an important factor affecting the crop root zone thermal characteristics. In this study, the total moisture content was accurately controlled. The influence of soil moisture on soil thermal conductivity mainly occurs through changing the heat transfer path of the soil. Before the moisture content rises to the critical moisture content, the water and air in the pores are replaced, and the solid‒gas heat conduction path decreases, while the solid‒liquid heat conduction path increases [43]. The heat conductivity of the crop root zone increases with increasing soil moisture content. Cai et al. also concluded that the soil thermal resistivity decreases significantly at the beginning of the gradual wetting of dry soil [17]. In addition, as soil heat capacity is also mainly determined by soil moisture content, there is a significant positive correlation between soil heat capacity and moisture content [44]. The same conclusion was reached in this study. Salomone et al. concluded that the critical moisture content of sandy loam is approximately 15%–20%. The moisture content of each soil layer under each treatment was less than the critical moisture content [43]. The thermal conductivity and heat capacity of the crop root zone were significantly positively correlated with the soil moisture content (Fig. 9). Notably, the HCR treatment could significantly increase the soil moisture content compared with other modified treatments, which may have been due to the increase in water retention and conductivity of the root zone soil by root irrigation with MWCNTs, making the saline farmland soil more stable [45]. Compared with applying MWCNTs alone, applying B. atrophaeus alone could significantly reduce the soil moisture content. Applying B. atrophaeus together with MWCNTs (BM and BR treatments) promoted a reduction in the soil moisture content. The combined application could play an active role in promoting crop growth, increasing crop water consumption, and reducing soil moisture content, thus reducing the thermal conductivity and heat capacity of the crop root zone.

The application of MWCNTs and B. atrophaeus also affects the spatial distribution of soil salt by changing the root-soil interactions, thereby indirectly affecting the crop root zone thermal characteristics. Although the influence of the soil salt content on thermal characteristics is less than that of the soil water content, its influence on thermal characteristics should not be completely ignored [46]. With increasing soil salt content, the electric repulsion force between soil particles decreases, and the force between adjacent soil particles changes from an electric repulsion force to an electric attraction force, which increases the contact between soil particles, the heat transfer path and the soil thermal conductivity [46,47]. The same phenomenon was also observed in the study of Malek et al. with increasing soil salt content, the thermal conductivity gradually increased [48]. This was also concluded in this study. The soil salt content was significantly positively correlated with the root zone thermal conductivity, and the root zone heat capacity also showed the same rule (Fig. 9). In the process of crop growth, the soil salt is driven by the difference in potential energy and accumulates in the direction of the crop roots along with the soil moisture, so the thermal conductivity and heat capacity of the rich root zone are higher than those of the poor root zone (Fig. 2, Fig. 3). The stronger the water absorption capacity of the crop roots, the stronger the aggregation effect, which is also the reason why the combined application of MWCNTs and B. atrophaeus (HBM and HBR treatments) significantly increased the lateral migration content of soil salt, which increased the thermal conductivity and heat capacity of the rich root zone to a certain extent. Moreover, the effect of MWCNTs on soil salt migration in the root zone was greater than that of B. atrophaeus, which may be due to the electric charge of MWCNTs themselves, which increased the electrical repulsion between soil particles, the soil salt migration path, and the salt migration rate in the soil, and significantly affected the crop root zone thermal characteristics [49]. In addition, the different longitudinal distribution patterns of the soil salt under the two soil salt content conditions were caused by a combination of upward salt migration with soil moisture and downward leaching with irrigation. The soil evaporation was the same in all treatments as assumed, the upward soil salt migration with soil moisture was mainly controlled by the level of soil salt content. The higher the total soil salt content was, the greater the upward migration of salt. In addition, under high salt conditions, the HBM treatment significantly increased the salt content of the deep soil compared with the other modified treatments. It is speculated that the presence of crop roots promoted the downward migration of soil salt and increased the thermal conductivity and heat capacity of the deep crop root zone to a certain extent. Therefore, the application of MWCNTs and B. atrophaeus can change the spatial distribution of soil salt, increase the soil salt content in the rich root zone and deep root zone, and increase the thermal conductivity and heat capacity of those zones.

The application of MWCNTs and B. atrophaeus also indirectly affects the crop root zone thermal characteristics by changing the growth of crop roots, changing the characteristics of soil particle size, affecting the contact degree between soil particles, and affecting the formation of continuous water film on the soil surface [50]. Under a poor root density (HCK treatment), crop roots will increase the contact area between soil particles, increase the heat transfer path, and increase the soil thermal conductivity. However, under a rich root density (LBK and LBM treatment), the influence of crop roots on the degree of soil particle contact and the soil particle size change is not significant [44,51]. The same conclusion was reached in this study (Fig. 9); the thermal conductivity of the crop root zone was significantly negatively correlated with the soil particle size and was significantly positively correlated with the specific surface area and the soil clay content. Some researchers have proven that the smaller the soil aggregate size is, the higher the soil clay content and the greater the soil thermal conductivity [52]. This explains why the HBM and HBR treatments reduced the thermal conductivity and heat capacity of the root zone. The combined application of MWCNTs and B. atrophaeus significantly reduced the content of soil clay particles under high salt conditions, the contact area of the soil particles, and the heat transfer path, and thus reduced the thermal conductivity of the crop root zone. However, the effect of applying these two new fertilizers alone on soil particle size was not significant, nor was the effect of changing soil particle size on the root zone thermal characteristics. Cai et al. proved that the thermal conductivity increases with increasing dry density and soil particle size [17]. In addition, the soil particle size distribution also affects the water-salt transport to a certain extent and indirectly affects the soil thermal characteristics [53]. Soil salt will make the soil particle size smaller, and the combined application of MWCNTs and B. atrophaeus will slow the trend of smaller soil particle sizes due to soil salt. For example, the LBR treatment reduced MN by 9.35% compared with the HBR treatment, while the CK treatment reduced MN by 12.00%, which increased the soil thermal conductivity to some extent. Therefore, the application of MWCNTs and B. atrophaeus alone had no significant impact on the soil particle size characteristics but alleviated the damage of soil salt to soil particles. The combined application of two new fertilizers (LBR treatment) under high salt conditions significantly reduced the soil particle size, increased the soil particle contact area, reduced the soil clay content, and indirectly increased the thermal conductivity and heat capacity of the crop root zone.

### 4.2 Direct influences

Moreover, applying MWCNTs and B. atrophaeus directly affected the thermal characteristics of the crop root zone by changing the soil properties. The high ionic strength in saline farmland soils can also cause MWCNTs to gather rapidly in the soil, weaken the transport of MWCNTs in porous media, and significantly change the soil properties in the root zone, which directly affects the crop root zone thermal characteristics [54,55]. Through this aggregation, MWCNTs applied by root irrigation can reduce the dry density of saline soil, improve the soil quality, and make the soil structure more stable [45], thereby reducing the thermal conductivity and heat capacity of the crop root zone. The thermal conductivity of MWCNTs is far greater than that of soil particles and moisture [56]; with root irrigation, the thermal conductivity of the root zone will increase, which may have been the reason for the significant increase in thermal conductivity and thermal diffusivity of the root zone of the CR treatment. Zhao et al. show that the higher the soil water repellency is, the smaller the thermal conductivity [57]. Because MWCNTs have a large porosity, large specific surface area, strong hydrophobicity and other characteristics, the MWCNTs applied by root irrigation are speculated to reduce the soil thermal conductivity by enhancing the soil water repellency. Moreover, applying MWCNTs and B. atrophaeus can also change the characteristics of the soil bacterial community and promote soil microbial activity [25,58]. With the movement of bacteria and microorganisms in the soil, a certain amount of biological pores will be produced to improve the soil porosity [59] and reduce the heat transfer path, thereby reducing the thermal conductivity and heat capacity of the root zone. In addition, compared with soil particles, soil organic carbon can store more moisture, promote the formation of soil aggregates, and stabilize pore structure, thereby improving soil heat capacity and assisting in soil temperature maintenance [60,61]. In this study, the soil organic carbon content was not significantly related to the crop root zone thermal characteristics, crop root characteristics or soil properties, which may have been because soil organic carbon content is related to crop growth and soil microbial activity, and its impact on the thermal characteristics of the root zone is generally indirect. This study did not focus on this, and there was no notable effect of applying MWCNTs and B. atrophaeus on the soil organic carbon content. The correlation among the root zone thermal diffusivity, soil properties and crop root characteristics was not significant, which may have been because it was significantly positively correlated with thermal conductivity and thermal capacity. The thermal diffusivity is proportional to thermal conductivity and inversely proportional to thermal capacity, so it was not significantly affected by those factors and requires further exploration.

In the past, there have been studies on the use of carbon-based materials to improve soil thermal properties, it has been proved that the medium fraction biochar has the ability to reduce soil thermal properties and proves the efficacy of biochar as thermal backfill [62]. In addition, soil thermal remediation technology is also widely used in engineering technology. Improving heating power, reducing water content and preventing surrounding water leakage are all beneficial to the soil heating process [63]. But these technologies are rarely used in farmland, and materials such as biochar cannot be directly used by crops. Thermal remediation technology is expensive and not suitable for farmland. Therefore, the use of carbon nanomaterials and strains in this study can not only regulate the thermal characteristics of crop root zones, but also promote the growth of crop roots, which has a positive effect on agricultural production.

## 5 Conclusion

In this study, the effects of applying MWCNTs and B. atrophaeus on the crop root zone thermal characteristics in saline farmland soils were investigated through pot simulation tests, and the response relationship among crop root zone thermal characteristics, soil properties and root characteristics was expounded.

The results showed that MWCNTs and B. atrophaeus could indirectly affect the crop root zone thermal characteristics by changing the growth of crop roots. The combined application of MWCNTs and B. atrophaeus could promote both fertilizers and achieve positive effects, promote the growth of crop roots, and significantly alleviate the adverse effects caused by soil salinization. With increasing soil depth, the thermal conductivity and heat capacity of the root zone gradually increased. The thermal conductivity and heat capacity of the shallow root zone were reduced due to the presence of crop roots, while the opposite result was found in the deep root zone. For example, the thermal conductivity of the 0–5 cm rich root zone in the MWCNT treatment was 0.8174 W m^−1^ ·K^−1^, and the thermal conductivity of the poor root zone was 13.42% higher than that of the rich root zone. The application of these fertilizers can also change the spatial distribution of soil moisture, soil salt, and soil particle size characteristics by influencing the root-soil interactions, indirectly affecting the crop root zone thermal characteristics. In addition, MWCNTs and B. atrophaeus can directly affect the root zone thermal characteristics by changing the soil properties. The effects of each treatment on the root zone thermal characteristics, soil properties and crop root characteristics under high salt conditions were more significant than those under low salt conditions. The higher the soil salt content was, the more obvious the adjustment effect of MWCNTs and B. atrophaeus on crop root zone thermal characteristics. The thermal conductivity and heat capacity of the crop root zone were positively correlated with the soil moisture content, soil salt content and soil particle specific surface area and negatively correlated with the soil particle size and fresh and dry weight of the roots but had no significant correlation with the soil organic carbon content. In this study, there was no significant correlation among the thermal diffusivity of the crop root zone, soil properties and crop root characteristics.

In summary, crop root characteristics are factors that cannot be ignored when exploring crop root zone thermal characteristics. The application of MWCNTs and B. atrophaeus can not only directly affect the root zone thermal characteristics of saline farmland soils by changing the soil properties of the crop root zone but also indirectly affect those characteristics by changing the crop root growth and root-soil interactions. This shows that MWCNTs and B. atrophaeus have ability to change the crop root zone thermal characteristics and adjust the temperature of the root zone in saline farmland, which helps crops and root zone microorganisms better adapt to the climate conditions of global warming, improve the availability of water resources in saline farmland, and lay a solid theoretical foundation for large-scale application on saline farmland in the future.

## Author contribution statement

Yutian Zuo: Performed the experiments; Wrote the paper.

Wenzhi Zeng; Jiesheng Huang: Conceived and designed the experiments.

Chang Ao: Contributed reagents, materials, analysis tools or data.

Haorui Chen: Analyzed and interpreted the data.

## Funding statement

Professor Wenzhi Zeng was supported by the 10.13039/501100012166National Key Research and Development Program of China [2021YFD1900805-03] and 10.13039/501100001809National Natural Science Foundation of China [52179039 & 51879196]. Professor Jiesheng Huang was supported by 10.13039/501100001809National Natural Science Foundation of China [51790533].

## Data availability statement

Data will be made available on request.

## Declaration of interest’s statement

The authors declare no competing interests.
