# Supplementary material for: Effects of multiwalled carbon nanotube and Bacillus atrophaeus application on crop root zone thermal characteristics of saline farmland
Source: Heliyon. 2023 Feb 8;9(2):e13510. doi: 10.1016/j.heliyon.2023.e13510 (PMC9947265; doi:10.1016/j.heliyon.2023.e13510)
Supplement: Multimedia component 1 [file mmc1.docx]

Table S1 Soil particle size characteristics during sampling

|  | MV (µm) | MN (µm) | MA (µm) | CS  (m^2^/cc) | Dia (µm) | Width | Clay (%) |
| --- | --- | --- | --- | --- | --- | --- | --- |
| HCK | 40.66±1.57e | 0.78±0.02c | 6.71±0.30d | 0.90±0.04a | 19.37±0.45d | 67.59±3.43cd | 8.46±0.51a |
| HCM | 39.74±4.63e | 0.79±0.06bc | 6.72±0.80d | 0.90±0.11a | 19.40±2.40d | 65.91±9.32d | 8.61±1.21a |
| HCR | 40.53±3.89e | 0.77±0.01c | 6.67±0.32d | 0.90±0.04a | 19.29±1.36d | 66.54±7.03d | 8.50±0.44a |
| HBK | 38.98±2.12e | 0.79±0.03bc | 6.80±0.25cd | 0.88±0.01a | 19.28±0.62d | 64.16±4.18d | 8.29±0.37ab |
| HBM | 43.12±3.68de | 0.78±0.01c | 7.00±0.31cd | 0.86±0.02ab | 20.71±1.51d | 72.42±8.55cd | 8.04±0.42b |
| HBR | 42.92±3.32de | 0.83±0.05b | 7.44±0.56c | 0.81±0.06b | 21.06±1.81cd | 70.42±5.92cd | 7.47±0.66b |
| LCK | 50.78±6.55b | 0.88±0.05a | 8.35±0.47ab | 0.72±0.04c | 23.40±1.19ab | 88.99±11.74ab | 6.45±0.47c |
| LCM | 49.90±5.05bc | 0.93±0.01a | 8.85±0.21a | 0.68±0.02c | 24.29±0.90ab | 86.42±11.00ab | 5.95±0.14c |
| LCR | 52.93±3.40ab | 0.90±0.01a | 8.69±0.62a | 0.69±0.05c | 24.73±1.03a | 91.67±3.02a | 6.18±0.62c |
| LBK | 51.44±3.46ab | 0.92±0.02a | 8.70±0.42a | 0.69±0.03c | 24.28±0.92ab | 88.91±6.78ab | 6.17±0.49c |
| LBM | 46.73±2.18cd | 0.90±0.01a | 8.17±0.38b | 0.74±0.03c | 22.74±1.62bc | 79.69±6.97bc | 6.62±0.34c |
| LBR | 55.94±5.41a | 0.92±0.01a | 8.80±0.24a | 0.68±0.02c | 23.27±4.11ab | 82.82±15.91abc | 6.11±0.21c |

Note: MV represents the mean volume diameter; MN represents the mean number diameter; MA represents the mean area diameter; CS represents the calculated specific surface area; Dia represents the particle size at a 50% unimodal distribution; Width represents the peak width of the particle size distribution; Clay represents the proportion of soil clay. All data are presented as the mean ± standard deviation. Lowercase letters show significant differences among the treatments (p < 0.05).
